# Herbicides do not ensure for higher wheat yield, but eliminate rare plant species

**DOI:** 10.1038/srep30112

**Published:** 2016-07-25

**Authors:** Sabrina Gaba, Edith Gabriel, Joël Chadœuf, Florent Bonneu, Vincent Bretagnolle

**Affiliations:** 1 Agroécologie, AgroSup Dijon, INRA, Univ. Bourgogne Franche-Comté, F-21000 Dijon, France; 2 LTER « Zone Atelier Plaine & Val de Sèvre », Centre d’Etudes Biologiques de Chizé, CNRS, F-79360 Villiers-en-Bois, France; 3 Avignon University, LMA EA2151, F-84900 Avignon, France; 4 Statistics, UR1052, 84143 Montfavet cedex, France; 5 Centre d’Etudes Biologiques de Chizé, CNRS & Université de La Rochelle, UMR 7372, 79360 Beauvoir sur Niort, France

## Abstract

Weed control is generally considered to be essential for crop production and herbicides have become the main method used for weed control in developed countries. However, concerns about harmful environmental consequences have led to strong pressure on farmers to reduce the use of herbicides. As food demand is forecast to increase by 50% over the next century, an in-depth quantitative analysis of crop yields, weeds and herbicides is required to balance economic and environmental issues. This study analysed the relationship between weeds, herbicides and winter wheat yields using data from 150 winter wheat fields in western France. A Bayesian hierarchical model was built to take account of farmers’ behaviour, including implicitly their perception of weeds and weed control practices, on the effectiveness of treatment. No relationship was detected between crop yields and herbicide use. Herbicides were found to be more effective at controlling rare plant species than abundant weed species. These results suggest that reducing the use of herbicides by up to 50% could maintain crop production, a result confirmed by previous studies, while encouraging weed biodiversity. Food security and biodiversity conservation may, therefore, be achieved simultaneously in intensive agriculture simply by reducing the use of herbicides.

Human food sources depend, directly or indirectly, on four main annual crops: wheat, barley, corn and rice^1^. Indeed the total economic value of annual crop production for human food has been estimated worldwide at around 1600 billion euros per year^2^, from 2005 FAO statistics. For centuries, weed control has been considered to be a critical issue and a limiting factor in crop production (review in^3^). Herbicides alone account for 37% pesticide active ingredients used worldwide^4^, and pesticides cost around 40 billion USD worldwide per year^5^, being said to save around 10% of losses to pests^6^, about 180 billion USD per year. Significant efforts have been made to increase the number of herbicides and their effectiveness^7^, review in ref. 8. However, as they generate large environmental costs, the use of herbicides, and more generally pesticides, has raised considerable concern with regard to their harmful consequences on ground and surface waters^9^, biodiversity^10^ and health^11^. Moreover, as many weed species are developing resistance to herbicides^12^,^13^ these species are becoming more difficult and expensive to control. Finally, it has recently been acknowledged that weeds in agro-ecosystems play an important role in maintaining ecosystem services (e.g., pollination: review in^14^; biological control^15^). Maintaining a balance between herbicide costs, weeds and crop production is, therefore, seen as the major challenge for agriculture in the future, from both economic and environmental viewpoints^4^.

There has recently been a general call to limit the use of herbicides at European and national levels^16^, either by reducing application rates, restricting the range of products (especially the most environmentally harmful) or using alternative management methods such as incorporating alfalfa in annual crop succession^17^ or sowing mixed crops^18^. However, farmers and scientists have expressed strong concern with regard to the potential negative indirect effects of a partial herbicide ban, since this may hamper food production^19^,^20^; see review in ref. 21. Despite many government incentives, the use of pesticides has not decreased significantly over the last ten years, either in Europe or in the US (see ref. 22). Through their expected effect on weeds (i.e., a major reduction in weed biomass), herbicides are implicitly thought to improve crop yields and so reducing the use of herbicides would indirectly reduce crop production. A strong relationship between herbicide use and crop yield is thus a critical expectation, although paradoxically, there is, at best, very little evidence to confirm such a relationship. Weeds may reduce the winter wheat yield by up to 23% on average worldwide, but actual loss due to weeds is less than 8% (Table 1 in ref. 3) and the adverse effect of weeds on crop yields is best established on organic farms^23^. Furthermore, although many studies of the effects of herbicides on weed populations are available, most were conducted many years ago (review in ref. 24), as most herbicides and active ingredients came onto the market prior to the eighties^8^. Moreover, almost all these studies were conducted on single species and in experimental conditions^24^,^25^; but see ref. 26). Therefore, the negative effect of weeds on crop yields has been modelled rather than tested empirically^27^,^28^,^29^.

Experimental and modelling studies usually ignore one further aspect: the farmers’ decisions and practices^30^,^31^. Although the application rate is usually recommended by agrochemical firms, the effectiveness of herbicides depends on the application mode (i.e. timing, dose), environmental conditions (the relative humidity can increase herbicide efficacy), the choice of active ingredient, depending on the observed or expected weed species, and the agricultural techniques used in combination. There is strong evidence that farmers behave in different ways in response to strong weed pressure^32^, although this has not been accurately quantified (but see ref. 33,34). There may be differences in the appreciation of the risk encountered for a given level of weed abundance^31^, in the technique to be used to deal with the situation (typically, between tillage and use of herbicide) and in the herbicide treatment (type of active ingredient, frequency and dose^30^). Although this has been studied for organic farming^23^,^30^,^31^, there is considerable uncertainty about the interaction between weed abundance in conventional fields, a farmer’s behaviour and decisions and the effectiveness of weed control by the herbicides^35^.

This study used empirical data on weeds, herbicide practices and winter wheat yields from 150 fields belonging to 30 farmers, to determine whether the use of herbicides improved yields and/or decreased weed abundance. As no clear relationships between herbicide use and weeds nor between yields and weeds were detected using standard statistical models, we modelled these relationships taking into account implicitly the effects of farmers’ behaviour and of environmental conditions on the effectiveness of weed management. Although farmers’ behaviour is usually taken into account in decision support systems^31^,^33^ and in mental models^30^,^32^,^36^ using data obtained from surveys of farmers, for this study a hierarchical Bayesian framework was developed^37^ which modelled farmers’ behaviour (*sensu lato*) as a parameter influencing the latent variable, λ, of the expected number of weeds per unit area. This parameter quantifies the farmers’ impact on the pairwise ‘crop yield-herbicides-weeds’ relationships. This method differed from the conventional statistical approach by assuming that a farmer’s behaviour (denoted η^R^_F_ and η^A^_F_ for weed richness and abundance, respectively) affect the crop yield-herbicide relationship through his own perception of weeds and weed control management strategies (e.g. timing of treatment). To include more realistic conditions in the model, a framework was developed to take account of the adaptive management by a given farmer to deal with the specific conditions encountered in his fields, by allowing a nested effect of field within farmer (η^R^_Ff_ and η^A^_Ff_ for weed richness and abundance, respectively) and also taking account of the differential effectiveness of herbicide treatments depending on the weed species, η^R^_Ffs_ (η^A^_Ffs_). We then analysed the interactive effects of farmers’s behaviour, for both η_F_ (η^A^_F_) and η_Ff_ (η^A^_Ff_), and the herbicide application rate on weeds, testing the hypothesis that herbicide treatment affected the abundance of weeds rather than species richness and targeted species (those thought to reduce the yields) rather than non-targeted species, using estimated values of η^R^_Ffs_ (η^A^_Ffs_).

## Results

### Herbicide application rate did not affect weeds or crop yields

**Figure 1:**
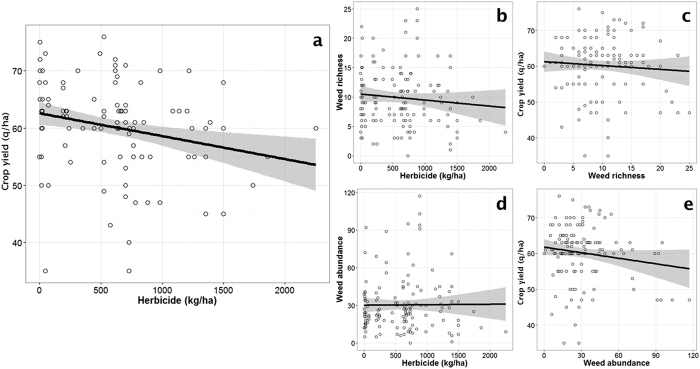
Pairwise relationships between crop yield (q.ha^−1^), weed richness (or weed abundance) and herbicide application rates. (**a**) Negative relationship between crop yield (q.ha^−1^) and herbicide applied (dose in kg.ha^−1^). The weed richness (**b**) and frequency (**d**) were not affected by herbicides. The crop yield was not significantly reduced by (**c**) weed richness or (**d**) frequency. The weed frequency is the sum of the weed presence in each quadrat. On each graph, the line and the smooth line represent the predictions of the linear mixed models and 95% confidence interval, respectively.

108 species were found over the 150 fields with an average of 9.46 species per field (range 0–25). All but one species of the six most commonly found were annual dicotyledons, i.e. *Polygonum aviculare* L., *Veronica persica* Poir., *Mercurialis annua* L., *Fallopia convolvulus* L. and *Galium aparine* L., with the exception of *Poa sp.* (annual monocotyledon). We first attempted to determine a positive relationship between the crop yield and the herbicide application rates, expressed as the total application rate over the cultivation period, using linear mixed models (with field nested within farmer as a random effect). The relationship between the crop yield and the herbicide application rate was actually negative (LMM: estimate = −0.0034 (SE = 0.0016), *F*_1,103.86_ = 4.933, *P* = 0.028; Fig. 1a; see Supplementary Material). Adding nitrogen as a covariate to control for the intensiveness of the crop production in the model did not change the result (ΔAIC <2 compared to the model without nitrogen input) and so nitrogen input was removed from the model. Furthermore, contrary to expectation, no significant relationships were observed between the herbicide application rate and either the weed frequency or the weed species richness (respectively *F*_1,116.66_ = 0.889, *P* = 0.347; Fig. 1b and F_1,131.62_ = 0.0006, *P* = 0.939; Fig. 1d) or between crop yield and species richness (Fig. 1c; *F*_1,132.31_ = 0.112, *P* = 0.738). There was a slight negative relationship between crop yield and weed frequency for the highest weed frequency, but the relationship was far from significant (Fig. 1e; F_1,118.62_ = 1.360, *P* = 0.246). Similar results (Fig. S2) were found when the level of herbicide application was described by a synthetic indicator: the Treatment Frequency Indicator (TFI, see Supplementary Material).

No evidence was found for any relationship between weeds, herbicide application rates and crop yield. One reason could be that farmers adapt their treatment strategy in order to keep the weed risk below a given threshold and guarantee a minimum yield^33^. However, the very high variances found in all pairwise relationships (Fig. 1) suggested testing an alternative scenario in which the variability in the farmer’s behaviour was so high that it masked any possible relationship. Farmer’s behaviour aggregates here what the farmer actually does (choice of active ingredients and number and timing of applications), interacting with the environmental conditions at the time of herbicide applications and the agricultural techniques used in combination with herbicides.

### Farmers’ behaviour affected the herbicide-weed relationship

**Figure 2:**
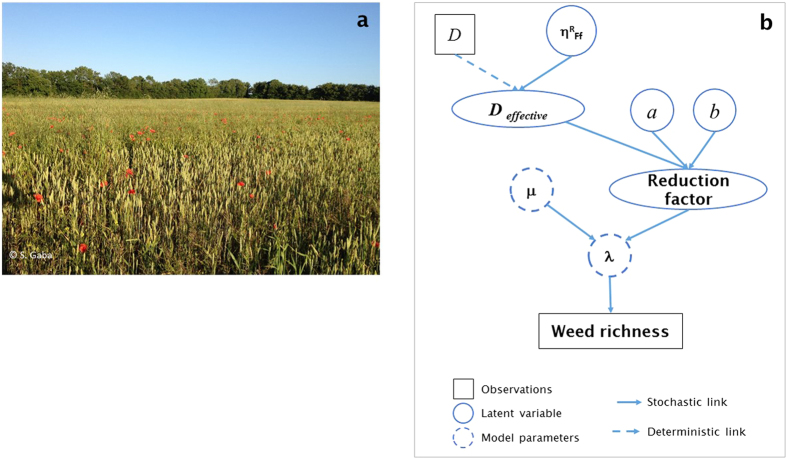
(**a**) Photographs of weeds in a winter wheat field (Sabrina Gaba photo credits). (**b**) Schematic representation of the Bayesian hierarchical model (Rich_field). It was assumed that, when no herbicide was applied, species richness among fields followed a Poisson distribution with mean μ. Herbicides reduced the species richness to an observed species richness λ (the number of species that survived treatment). The reduction was a function of three parameters: *a* was the scaling factor, *b* was the shape factor describing the concavity of the reduction curve and *D*_*effective*_ was the effectiveness of the herbicide depending on *D* and η^R^_Ff._ *D* was the observed herbicide application rate and η^R^_Ff_ was a parameter quantifying farmer’s effect on the effectiveness of the treatment in each of his field. Except for *D*, all parameters and latent variables were estimated from the observed data.

**Figure 3:**
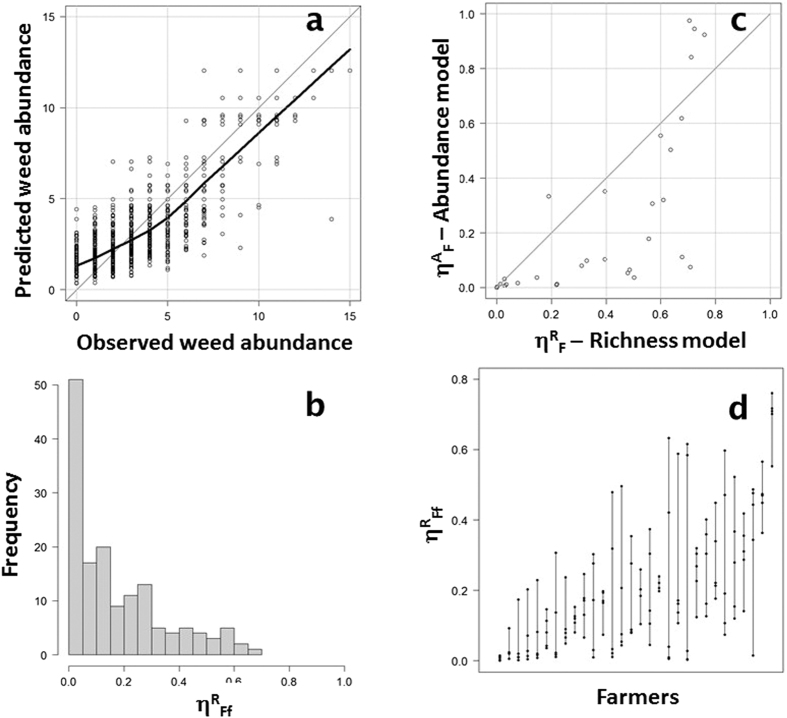
(**a**) Weed abundance estimated during the parameter estimation procedure and weed frequency (sum of weed presence in each quadrat) show a good fit. (**b**) η^R^_Ff_ estimated in the weed richness model (Rich_field) was plotted against the η^A^_Ff_ estimated in the weed estimated abundance model (Ab_field). If the effectiveness of the herbicide on weed richness and abundance was similar, the dots should fall on the y = x line. (**c**) Dispersion of η^R^_Ff_ across fields (Rich_field). A zero value indicates that the herbicide treatment did not have an effect. (**d**) Representation of the high variability of the effectiveness of treatment between farms and between fields farmed by the same farmer. The dots show the effectiveness of the treatment η^A^_Ff_ per farmer which are classified by increasing η^A^_F_.

Hierarchical Bayesian models were used to model the effect of herbicides on weed richness and abundance (Fig. 2; Table 1) taking into account the variability in the farmer’s behaviour. Such variability was introduced to model either a simple farmer effect (η^R^_F_ and η^A^_F_) assuming a similar effect across the five fields farmed by the farmer, or with variability between fields for a given farmer, which was modelled as a nested effect at field scale within a farm (η^R^_Ff_ and η^A^_Ff_) (Table 1). The first set of models (Table 1) assumed that the effectiveness of herbicides did not vary with weed species (although all species abundances were modelled separately). The model fit was tuned by comparing weed richness or abundance as estimated by the model output with the observed values. The model with the nested effect at field scale within a farm (η_Ff_) explained the variability in weed species richness much better (Rich_field: DIC = 31600; Fig. S3) than the model with only the farmer effect (η_F_; Rich_farm DIC = 32590). This model also explained the weed species richness much better than the model without any farmer effect (Rich_base: DIC = 34200). Similar results were found for weed estimated abundance (Ab_field: DIC of the model with η^A^_Ff_ = 7069, Ab_farm: DIC of the model with η^A^_F_ = 6142 and Ab_base: DIC of the model without any effect = 5508; Fig. 3a). Estimated parameters are given in Table S3.

η^R^_F_ (η^A^_F_) and η^R^_Ff_ (η^A^_Ff_) are surrogates for the effectiveness of treatment and vary between 0 and 1, a value of 1 being the effectiveness expected if weed control were complete. There was a strong farmer identity effect on the effectiveness of the weed control treatment (Fig. 3b): the farmers’ effect appeared to depend on the field (see variation of η^A^_Ff_ over the five fields farmed by each farmer in Fig. 3d), as already shown based on surveys of farmers^32^,^34^. This suggests that farmers either adapted their management at field level, or possibly that the effectiveness differed between fields because of exogenous factors (e.g. meteorological conditions are known to affect the effectiveness of herbicides^38^). Herbicide management options varied for 19 farmers out of the 30 (63.3%) in our dataset suggesting that farmers changed their management practices to some extent depending on the field. Only one third of the 30 farmers applied exactly the same herbicide treatment to each of their five fields and so the differences between fields is likely to have been due to the effectiveness of the treatment rather than the herbicide used. Furthermore, there was only weak and non-significant correlation between η^R^_Ff_ (η^A^_Ff_) and the farmers’ weeding strategies, described by the diversity and date of introduction of products applied as well as the number of tank-mixed commercial herbicides (*SM*, Fig. S5 and Table S5).

Most η^R^_Ff_ values in the weed richness model were close to 0 with a maximum of 0.65 (Fig. 3b), indicating significant discrepancies between the expected effect of the herbicide treatment at a given strength and the effect observed on weed richness. The asymmetric distribution (Fig. 3b) of η^R^_Ff_ suggested that most herbicide treatments had almost no effect and that, for almost all the farmers, the treatment did not reduce either weed richness or weed abundance, i.e. η^A^_Ff_ was close to 0, in at least one of their fields (Fig. 3d). 64.5% of η^R^_Ff_ estimates for the richness model and 60% of η^A^_Ff_ estimates for the abundance model were below 0.2 (Fig. S3C). In addition, η^R^_Ff_ estimates for the richness model (Rich_field) were generally lower than η^A^_Ff_ estimates for the abundance model (Ab_field, Fig. 3c), suggesting that herbicides tended to be generally more efficient at controlling total weed richness than weed estimated abundance.

### Herbicides were effective for controlling rare species but did not control abundant species

**Figure 4:**
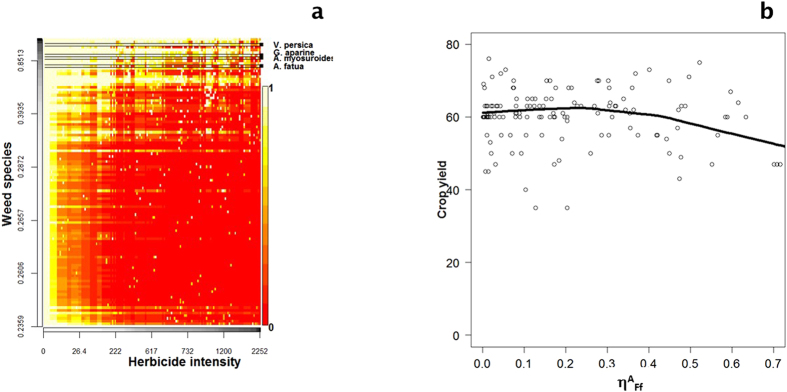
(**a**) Weed species survival rate depending on the herbicide application rate. On the y-axis, the weed species are classified from low to high abundant species, the abundance being estimated in absence of herbicide applications. Weed Red values indicate high mortality rate (survival rate close to 0) and high survival rates are indicated by light yellow or white values. The higher mortality rates (red values) are observed for the rare species. Four of the most noxious species are: *Veronica persica*, *Galium aparine*, *Alopecurus myosuroides* and *Avena fatua*. (**b**) Relationship between crop yield (q.ha^−1^) and the effectiveness of the treatment η^A^_Ff_. The LOESS regression is shown by a line.

To give more realistic conditions and models, we then relaxed the assumption of identical effect of herbicides on weed species, and assumed that the effectiveness of herbicide varied with species identity, i.e. η^A^_Ffs_ estimates the effectiveness of herbicide treatment on a given weed species in each field for each farmer (model Ab_spec in Table 1). Determining the probability of weed species survival in relation to the amount of herbicide applied, and depending on the relative weed abundance, showed that herbicides were very effective at suppressing rare weeds (i.e. the less abundant species in absence of herbicides) but less effective at suppressing the most abundant weeds (Figs 4a and S5). In a small number of cases, herbicides reduced the survival of abundant species (upper part of Fig. 4a) but only when high doses were applied (i.e., upper 90% quartile). The survival probability profiles of the most abundant species differed from species with lower abundance, again indicating that herbicide was not a primary factor in controlling the most abundant weed species (top part of Fig. 4a). Herbicides also failed to control four of the most noxious weed species identified by farmers in the study site (see methods section, and ref. 39). Although herbicides failed to control abundant, targeted noxious weeds, we tested whether fields where treatment was the most effective had the highest wheat yields. This was not the case, since there was no relationship between the effectiveness of herbicide treatment (η^R^_Ff_) and wheat yield (Fig. 4b).

## Discussion

The main purpose of this study was to determine whether decreasing the amount of herbicide used would significantly reduce yield owing to an increase in weed richness and/or abundance, as has frequently been suggested^20^; see review in ref. 21. However, using a dataset of 150 fields, there was no correlation between weed richness or frequency and winter wheat yields. Furthermore, no correlation was found to indicate that the herbicide application rate had an effect on weeds or on yield. Taking account of the possible role of farmers and environmental conditions in the effectiveness of treatment, the results suggested that many treatments were ineffective (Fig. 3c), probably accounting for the lack of effects. Even where treatment was effective, however, there was no correlation between the effectiveness of treatment and yield (Fig. 4b). Even though herbicide application rates had no effect on weed estimated abundance, including targeted species, or on yield, the results suggested that the only tangible effect of herbicides was on less abundant weed species, which were not targeted by farmers. The validity and robustness of this approach is discussed below. The findings are compared with available literature and some consequences of the study with regard to pesticide use and biodiversity management in farmlands are described.

The crop yield losses resulting from a reduction in pesticide use is generally quantified without taking account of the effect of farmers’ decisions (e.g. ref. 40). Our study used Bayesian Hierarchical Models with a latent variable which models the farmer’s behaviour (including, e.g., application mode, choice of active ingredient, cropping systems, farmer’ belief and perception) interacting with environmental conditions. Bayesian and Markov hierarchical models with hidden state variables to allow for human behaviour have commonly been used^41^ for decision models^42^ and for policy-making because they can realistically predict human behaviour^43^ or easily accommodate underlying environmental attitudes^44^. In this study, the modelling approach relied on several strong assumptions. Firstly, it was assumed that weed species were randomly distributed in a given area, with a Poisson distribution. This assumption was used to estimate the average number of species to be expected in a field where herbicide had been applied and compare this estimated value with the observed value. There is some evidence that weed species are distributed randomly in farmland areas or at least that random assemblage of weeds (*sensu* neutral model^45^) cannot be disregarded. For instance, in the same study site^46^ found that weed communities in organic farms were best explained by mass effect metacommunity models, and ref. 47, also in the same study site, found that weed functional diversity differed very little from random assemblage, in particular in winter wheat. We also assumed that the abundance of each species also had a Poisson distribution, although this is a much more conventional, less controversial assumption^48^,^49^, and was a good predictor of its cover. Indeed farmers could respond to cover, and not to abundance which could also explained the lack of relationship between herbicides and weed estimated abundance. Secondly, the effect of the herbicide application rate on weed richness (abundance) was expressed using a non-linear function. We made this assumption in the model structure to ensure that the estimated value of weed richness (abundance) decreased with decreasing herbicide application rates and remained positive (or null). A reduction factor (Fig. 2c) was used to describe how farmers’ management decisions affected the effectiveness of herbicides, i.e. it was assumed that herbicides were not fully effective with a difference between the observed richness (or abundance) and the expected richness (or abundance) for perfect effectiveness of the herbicide treatment. Thirdly, the herbicide application rate was described using two different indicators, the total dose of herbicides and the TFI, which describe complementary aspects of weed control treatments. The results were similar for either indicator (details are given in *SM*). Finally, although in previous studies of weeds, herbicides and yields the sample size was often limited (e.g. 15 farms in ref. 34; 16 farms in ref. 30; 10 trials in ref. 50), our sample size was reasonably large (30 farms and 150 fields), although it was limited to a single geographical area and a single year. Investigating the weed-crop yield relationship over several years would allow quantifying the effect of climate on weeds a well as crop biomass production, and the output of their interactive relationship. In addition, this study considered only conventional farming. Despite it is the most common farming system in developed countries, it would be of great interest to include alternative farming systems such as organic farming in this analysis to explore the effect of mechanical weeding on the weed-crop yield relationship (e.g., organic farming and Agri-Environmental Schemes in ref. 9, which used the same data set for France). This obviously requires further analyses carried out in different areas, for different farming systems and over several years.

Despite repeated claims that weed density lowers yields (e.g. review in ref. 24), the evidence is less conclusive than usually claimed^51^. In an extensive review^24^ established that at least 30 species of weeds reduce wheat yield to varying degrees (ranging from a few % up to 75%) and at a highly variable threshold of number of seeds or plants/m^2^. However, extremely few studies have investigated this effect at community level (none in ref. 24 for instance)^52^ studied the long-term effects of applying full and half doses of herbicide on 10 fields: compared to a control, full and half doses increased the proportion of difficult-to-control weed species significantly in half of the sites, while crop yields were actually higher in some sites when using half doses. Many other studies have demonstrated that doses can be reduced by 50% or even more compared to the recommended dose without detectable loss of yield^52^,^53^, increase in weeds^54^ or both (review in ref. 21). Indeed, without crop being present, weed control was at least 70% effective in 50% of the studies, even when the herbicide application rate was only 20% of the recommended rate, whereas in conjunction with crop cultivation, no detectable effect was found with up to 50% reduction in herbicide use compared to the recommended doses^21^. Furthermore, using experimental data from the literature^55^ found that wheat has the highest competitive ability among 26 crops against weeds. Consequently, weed competition may have little effect on winter wheat (certainly lower than on other crop species), which questions the use of large amounts of herbicide in winter wheat cropping systems.

Since the introduction of herbicides (in the 50s^8^), weeds have become a secondary problem for farmers and were no longer considered a decisive factor in the design of farming systems^34^. For decades, herbicides allowed farmers to hope for totally weed-free fields. Nowadays, maximum weed control has been shown to be unnecessary, even to achieve high yields or income^53^,^55^,^56^. Besides providing new evidence, this study suggested that herbicide use did not increase yields and affected rare species (i.e. species at low abundance in absence of herbicide application) rather than common weed species and non-targeted species rather than noxious species. The analysis focused solely on wheat, which is the most important crop in the world (in terms of area cultivated), and weeds are the most important pest group in wheat production worldwide^3^. We believe, therefore, that the results suggest that a reappraisal of how herbicides affect yields of major crops is needed.

If reducing herbicides by more than 50% would increase biodiversity and reduce contamination of water and risk to health, with an undetectable effect on yield, it would further increase farmer’s income (i.e. lower costs for farmers for equivalent crop yields). Despite these clear advantages, farmers are reluctant to reduce herbicide use: for instance, integrated pest management (IPM) has long been promoted by experts^22^,^57^ for economic and environmental reasons but is still seldom used. It has been suggested that farmers continue to use herbicides despite their effects on environmental sustainability, as well as farmers’ health, because of their awareness of the delayed risks of lower weed control, with increasing seedbank density^32^. Alternatively, farmers’ use of herbicides may be rooted in a market system that encourages the adoption of biophysically unsustainable techniques^11^: these may lower current costs and boost yields in the short term but eventually lower yields and raise production costs in the longer term^58^. Agricultural practices tend to continue to apply such systems once they have been adopted even though they are unsustainable^58^,^59^. All the possible explanations of our results call for mid-term (>4 to 6 years) experimental studies that explicitly incorporate the farmer’s behaviour (weeding practices, perceptions, attitudes to weeds) thus requiring interdisciplinary research (socio-economic, agricultural and ecology sciences). These experiments could be implemented in different countries where wheat is an important crop.

To ensure food security while conserving biodiversity in intensive agriculture, government policies have often targeted a combination of changes in herbicide use with increased diversification in crop rotations, as well as the use of IPM or organic farming^13^,^22^. We argue here that it is perhaps far easier merely to reduce the use of herbicides.

## Materials and Methods

### Study area and sampling design

In 2007, 30 farms were selected in the LTER “Zone Atelier Plaine & Val de Sèvre” (Supplementary Material), with no particular spatial or agronomic design, except that organic farms or farms engaged in agri-environmental schemes (AES) were *a priori* excluded (but see details in ref. 10). None of the 30 farmers used mechanical weeding methods for weed control. The general characteristics of the farms are presented in Table S1. For each farm, five winter wheat fields were selected in consultation with the farmer, with no *a priori* selection. The fields were distributed throughout the study site (Fig. S1). All fields sampled from different farms were at least 1 km apart.

### Survey of farmers and herbicide treatments

Information about crop yields and farming practices (pesticide and fertilizer use, ploughing and mechanical weed control system) and general information about the farm (number of crops, proportion of land covered by AES, field size) was collected by means of a questionnaire sent out to all participating farmers. The response was 98% representing 30 farms. Herbicide use was described by the name and the concentration of each of the active ingredient and the day or week of application. Herbicides were further classified as monocotyledon specific, dicotyledon specific or broad spectrum. Crop yields were not available for 3 of the 30 farms.

### Weed surveys

Botanical surveys were carried out once during the flowering to milk-ripening stage of winter wheat, in spring/summer 2007^10^. For each of the 150 fields, surveys were carried out in ten quadrats (4 m^2^) at 10 m intervals in line from the border of the field toward the centre, perpendicular to the tracks made by farm machinery within the field. The first quadrat was 20 meters from the edge of the field. For each quadrat, weed species were recorded as either present or absent, irrespective of the number of individual plants, giving a list of species present in each quadrat.

### Statistical analysis of the relationships between crop yield–herbicides and crop yield–weeds

The relationship between crop yield and herbicides was analysed using a linear mixed model (LMM) with the farmer as random effect and with and without nitrogen input as a co-variable. Two indicators were used for the amount of herbicide applied: the total application rate and the treatment frequency index (*SM Materials and Methods*). LMM were analysed with a type III analysis of variance with Satterthwaite approximation for degrees of freedom. A model selection procedure based on Akaïke Criterion (AIC^60^) was performed to determine the effect of nitrogen input. The same procedure was applied to analyse the relationships between crop yield and weed richness (abundance). All analyses were performed using the R “LmerTest package”^60^,^61^

### Modelling farmers’ behaviour in the herbicide-weed relationships-Herbicide-Weed species richness model

In order to account for the high variability in the herbicide-weed richness relationship (LMM described above)^62^, hierarchical Bayesian models were used (Fig. 2c). It was assumed that the number of weed species in a given area, i.e. species richness, followed a Poisson distribution with mean *μ* when no treatment was applied. It was also assumed that herbicide treatment reduced the mean number of species and, therefore, λ, the species richness expected in a given area when a treatment was applied, was modelled as a Poisson distribution of mean μ/(1 + *a*D)^b^ where *D* is the amount of herbicide applied, *a* is a scale factor and *b* is a shape factor describing the concavity of the reduction after the application of the herbicide. The non-linear function of *D* allows the species richness to tend to zero as *D* becomes large, and to equal *μ* when no herbicide is applied (*D* = 0). A second model took account of farmers’ behaviour on the effectiveness of chemical weed control. A parameter η^R^_F_ was used to describe the effectiveness of the treatment as a function of the farmer, λ being modelled as a Poisson distribution with mean μ/(1 + *a*η^R^_F_D)^b^. All fields of a given farm, F, shared a common farmer effect, η^R^_F_. A third model included the farmer effect at field scale with a factor η^R^_Ff_ for field *f* belonging to a farm *F* as η^R^_Ff_ = η^R^_F_ η^R^_f_. The best of the three models (without η^R^_F_, with η^R^_F_ and with η^R^_Ff_) was selected based on the deviance information criterion (DIC) which is a hierarchical modelling generalization of the AIC^63^.

### Herbicide-Weed abundance model

No relationship was observed between herbicide use and abundance using LMM. Consequently, hierarchical models similar to those for the herbicide-weed species richness were built but, at the last step the species abundance was estimated using the presence-absence data (see details in *Estimating weed abundance*). The initial model assumed that herbicides had a similar effect on all weed species in the field and two models were built: one considered the same effect of the farmer’s decision in all his fields λ_s_ = μ_s_/(1 + *a*η^A^_F_*D*)^b^ and the other modelled the effect of the farmer’s behaviour at field scale λ_s_ = μ_s_/(1 + *a*η^A^_Ff_*D*)^b^. In both models, λ_s_ was the average number of plants of a given weed species *s* in a given area. The second step was to build a more realistic model, λ_s_ = μ_s_/(1 + *a*_s_η^A^_Ffs_*D*)^bs^, which considered that the effect of the herbicide depended on the weed species *s*.

### Estimating weed species abundance and survival rate

The weed abundance at field scale was estimated by assuming that weed abundance follows a Poisson distribution and that the probability of finding at least one plant in an area W(4000 m^2^) was: 1 − *exp*(μ_s_W/(1 + *a*η^A^_F_D)^b^) (see *SI Materials and Methods* for further details). We measured the survival of a species as the probability to observed one individual in W. For a species *s* with abundance intensity λ_s_, its survival rate was therefore e^−Wλs^ under the assumption of Poisson distribution of the individuals of this species.

### Estimating the model parameters

The Bayesian posterior distributions for each of the model parameters, including uncertainty due to variability in the data and the uncertainty of prior information, were approximated using Monte Carlo–Markov chain (MCMC) methods with prior information for the parameters (μ, a, b and η^R^_F_/η^R^_Ff_/η^R^_Ffs_ (η^A^_F_/η^A^_Ff_/η^A^_Ffs_)). The following priors were used: a Gaussian distribution N(0, 10) for log(μ), log(*a*) and log(*b*), so that *a*, *b* and μ follow log-Gaussian distributions, ensuring that μ, a and b were strictly positive and a non-informative uniform distribution U(0, 1) for η^R^_F_ (η^A^_F_), η^R^_Ff_(η^A^_Ff_) and η^R^_Ffs_(η^A^_Ffs_). 20,000 iterations were run with three independent chains in the MCMC procedure. For each chain, the first 10,000 iterations were discarded. After this “burn-in” period, inferences were derived from a sample of 20,000 iterations. Modelling was performed using Winbugs^63^ under R with the BRugs package^64^. Both the convergence of MCMC chains using Gelman-Rubin convergence statistic^65^ and the performance of the estimate (ESM Fig. S5A,B) were assessed^66^.

## Additional Information

**How to cite this article**: Gaba, S. *et al*. Herbicides do not ensure for higher wheat yield, but eliminate rare plant species. *Sci. Rep.* **6**, 30112; doi: 10.1038/srep30112 (2016).

## Supplementary Material

Supplementary Information

## Figures and Tables

**Table 1:** Description of the Hierarchical Bayesian models.

	Identical effect of herbicides on weed species			The effectiveness of herbicide varieswith species identity
	No farmer’s behaviour	Effect of farmer’sbehaviour at the farm scale	Effect of farmer’s behaviour at the field scale	
Weed richness	Rich_base	Rich_farm	Rich_field	
	λ = μ/(1 + *a*D)^b^	λ = μ/(1 + *a*η_F_D)^b^	λ = μ/(1 + *a*η_Ff_D)^b^	
Weed abundance	Ab_base	Ab_farm	Ab_field	Ab_spec
	λ_S_ = μ_S_/(1 + *a*D)^b^	λ_S_ = μ_S_/(1 + *a*η_F_D)^b^	λ_S_ = μ_S_/(1 + *a*η_Ff_D)^b^	λ_S_ = μ_S_/(1 + *a*_*S*_η_Ffs_D)^bS^

“Rich” and “Ab” indicate the models used with weed richness and estimated abundance, respectively. λ is the species richness (abundance) among fields and follows a Poisson distribution with mean μ. *a* is the scaling factor, *b* is the shape factor describing the concavity of the reduction curve, *D* is the herbicide application rate and η^R^. (η^A^.) is a parameter quantifying farmer’s effect on the effectiveness of the treatment in his farm (η^R^_**F**_ or η^A^_**F**_) or in each of his field (η^R^_**Ff**_ or η^A^_**Ff**_). In the “Ab_spec” model, the effectiveness of the herbicides varies with species identity. Except for *D*, all parameters and latent variables were estimated from the observed data.
