# Supplementary material for: Herbicides do not ensure for higher wheat yield, but eliminate rare plant species
Source: Sci Rep. 2016 Jul 25;6:30112. doi: 10.1038/srep30112 (PMC4958924; doi:10.1038/srep30112)
Supplement: Supplementary Information [file srep30112-s1.doc]

**Herbicides do not ensure for higher wheat yield but eliminate rare plant species**

**SM Materials and Methods**

Sabrina Gaba^1,2,*^, Edith Gabriel^3^, Joël Chadœuf^4^, Florent Bonneu^3^, and Vincent Bretagnolle^2,5^

^1^ Agroécologie, AgroSup Dijon, INRA, Univ. Bourgogne Franche-Comté, F-21000 Dijon, France ^2^ LTER « Zone Atelier Plaine & Val de Sèvre », Centre d'Etudes Biologiques de Chizé, CNRS, F-79360 Villiers-en-Bois, France

^3^ Avignon University, LMA EA2151, F-84900 Avignon, France

^4^ Statistics, UR1052, 84143 Montfavet cedex, France

^5^ Centre d’Etudes Biologiques de Chizé, CNRS & Université de La Rochelle, UMR 7372, 79360 Beauvoir sur Niort, France

^*^ Corresponding author: Sabrina Gaba [sabrina.gaba@dijon.inra.fr](mailto:sabrina.gaba@dijon.inra.fr)

**Study area.** The study site is the “Zone Atelier Plaine & Val de Sèvre” (http://www.za.plainevalsevre.cnrs.fr/), a long term ecological research site (LTER) of about 450 km² located in central western France, in the south of Deux-Sèvres district in the Poitou-Charentes region (46.23°N, 0.41W; Fig. *S1*). It is an agricultural landscape dominated by intensive cereal crop production (44% winter cereals, most of which are winter wheat varieties, 12% rape seed, sunflower or maize, and 14% meadows and alfalfa, 4% woods and villages; all data from 2010 season), with an average field size of 4-5 ha.

**Relationships between herbicide use, weeds and yield using standard statistical models.** Two indicators were used for the herbicide application rate: the total dose of herbicide and the Treatment Frequency Indicator (TFI). The dose was the sum of all the doses of herbicides used over the cultivation period. TFI was the sum of the dose applied of each herbicide normalized to the national recommended dose: TFI = ∑_T_ AD_T_/RD_T_, where T is the index of herbicide product, AD is the applied dose per hectare and RD is the recommended dose per hectare (1). This includes all the pesticide treatments applied in a given crop field (except for seed treatment), with the doses normalized by the national recommended doses in order to take account of the intensities of the treatments into account. As the vegetation was sampled and questionnaires were sent out in 2007, the TFI was calculated using the recommended doses for 2007 (2). Similar results were observed for with both indicators (Table *S3* and Fig. *S3*).

**Evaluation of the herbicide-weed models.** The ability of the model to estimate the species richness was assessed. Firstly, the various components of the model λ ~ Pois (μ/(1+a η^R^_F_ D)^b^) were estimated using the data and the expected species richness λ_1_ was estimated for each field. Secondly, the predicted number of species for each field, N_1_, was drawn from a Poisson distribution for the expected species richness λ_1_. The model was then fitted to these predicted points. Thirdly, the expected species richness λ_2_ was estimated using the predicted data and the new predicted number of species for each field, N2, was drawn from the Poisson distributions for the expected species richness. The second level predictions of the expected species richness and the predicted number of species for each field λ_2_ and N_2_ were then compared with the first level predictions λ_1_ and N_1_. The same procedure was applied to evaluate the model for weed species richness at quadrat level incorporating the effect of farmers’ behavior at field scale i.e. η^R^_Ff_. These two evaluations indicated that the method provided good estimates of weed species richness both with η^R^_F_ (Fig. S4) and η_Ff_ (data not shown). The model also gave a good estimate of the expected species richness λ_2_ from data based on λ_1_ (Fig. *S4A*; Kendall correlation test: rho=0.725, *P* <0.0001, *n*=150) and the predicted number of species present (Fig. *S4B*; Kendall correlation test: rho=0.355, *P*<0.0001, *n*=150). The correlation coefficient for the predicted number of species present was lower owing to the variability described by the Poisson function.

**Evaluation of weed abundance estimates**. Two procedures were used to estimate species abundance. An empirical procedure consisting in summing, for each field, the species present in the ten quadrats was used to analyze yield changes with abundance (Figs. 1*D*-*E*) and for checking the suitability of the the abundance model. The second procedure consisted in creating an abundance model for each species. For each species, the probability of finding at least one plant of the species in a given quadrat was
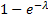
 where λ is the expected number of plants in the species in a quadrat. The expected number of plants in a quadrat was estimated at the same time as estimating the other parameters of the model (i.e. λ = μ/(1+*a* η^A^_Ff_ D)^b^ described above), with the same values for *a*, *b* and η^A^_Ff_ for all species. Since the quadrat observations were independent, the parameters were estimated using a Bayesian approach by computing the probability of the observations as the product over the quadrats of the probabilities of finding 0 or at least one plant in a quadrat. The total species abundance was then estimated as the sum of the species abundance.

**Farmers’ weeding strategies.** Three variables were used to describe the farmers’ strategy for herbicide use: *diversity*, *complexity* and *date of introduction*. *Diversity* measured the variability of herbicide use i.e. commercial herbicide preparations and doses, in the fields of a given farmer. It was assumed that herbicide use was diverse when the combination “commercial herbicide preparations and doses” differed in at least three of the five fields. Herbicide use was not diverse when the combinations were applied on at least four fields out of five on a given farm. Herbicide use was labeled as diverse on 13 out of 30 farms. *Complexity* was based on a score using the number of tank-mixed commercial herbicide preparations. The higher the number, the higher the complexity taking into account the presentation and any specific instructions linked to the use of the commercial herbicide preparation. Tank-mixed adjuvants were not taken into account. It was assumed that herbicide use was complex if at least three commercial herbicide preparations were tank-mixed in one field. Treatments were complex for 25 fields. The *date of introduction* of the herbicide used was based on the date when the active ingredients came on the market and was broken down into three classes: “recent”, “old” and “intermediate”. Recent herbicide preparations were those in which at least one active ingredient was still under patent. Old herbicide preparations were those in which all active ingredients came on the market before 1990. Other preparations were classified as intermediate. When at least one recent herbicide preparation had been used on a field, the treatment for the field was labeled as ‘recent’. When only old herbicide preparation(s) had been used on a field, the treatment for the field was labeled as ‘old’. In all other cases, the treatment for the field was labeled as ‘intermediate’. 50, 50 and 46 fields had been treated with “recent”, “intermediate” and “old” herbicides, respectively (four fields had not been sprayed with herbicides).

The relationship between the farmers’ strategy and η^A^_Ff_ was analyzed to determine whether the effectiveness of the herbicide treatment could be related to the complexity, diversity or date of introduction. The results for η^A^_Ff_ are presented in Figs. *S5A-C*. There was no significant relationship between the three variables, suggesting an absence of relationship between the herbicide strategy and the herbicide’s effectiveness. Similar results were found for η^R^_Ff_ (data not shown).

**References**

1. Pingault, N., Pleyber, E., Champeaux, C., Guichard, L. & Omon, B. (2009) Produits phytosanitaires et protection intégrée des cultures: l’indicateur de fréquence de traitement (IFT), Notes et Etudes Economiques, Ministère de l’agriculture et de la pêche, No 32. (Mars 2009). http://www.agreste.agriculture.gouv.fr/IMG/pdf_nese090332A3.pdf (accessed, April 2012).
2. ACTA (2007) Index phytosanitaire *43ème Edition*. Action Coordonnée des Techniques Agricoles, 149 rue de Bercy, 75955 Paris, France.

**Figure S1**: **Study site in the LTER “Plaine & Val de Sèvre” western France (**[**http://www.za.plainevalsevre.cnrs.fr/**](http://www.za.plainevalsevre.cnrs.fr/)**). The maps were created by Vincent Bretagnolle using ArGIS software 9.1 (https://www.arcgis.com/features/).**

**
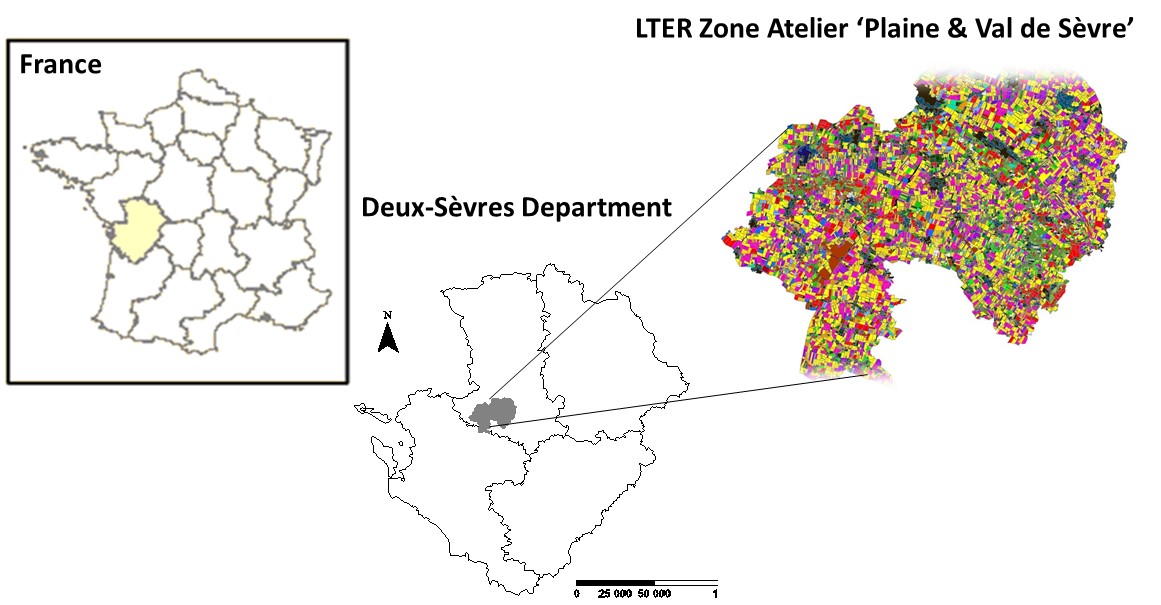
**

**Figure S2**: **Relationship between (a) crop yield (q/ha) and herbicide application rate, (b) herbicide application rate and weed species richness and (c) herbicide application rate and weed abundance. Herbicide application rate is represented by TFI.**


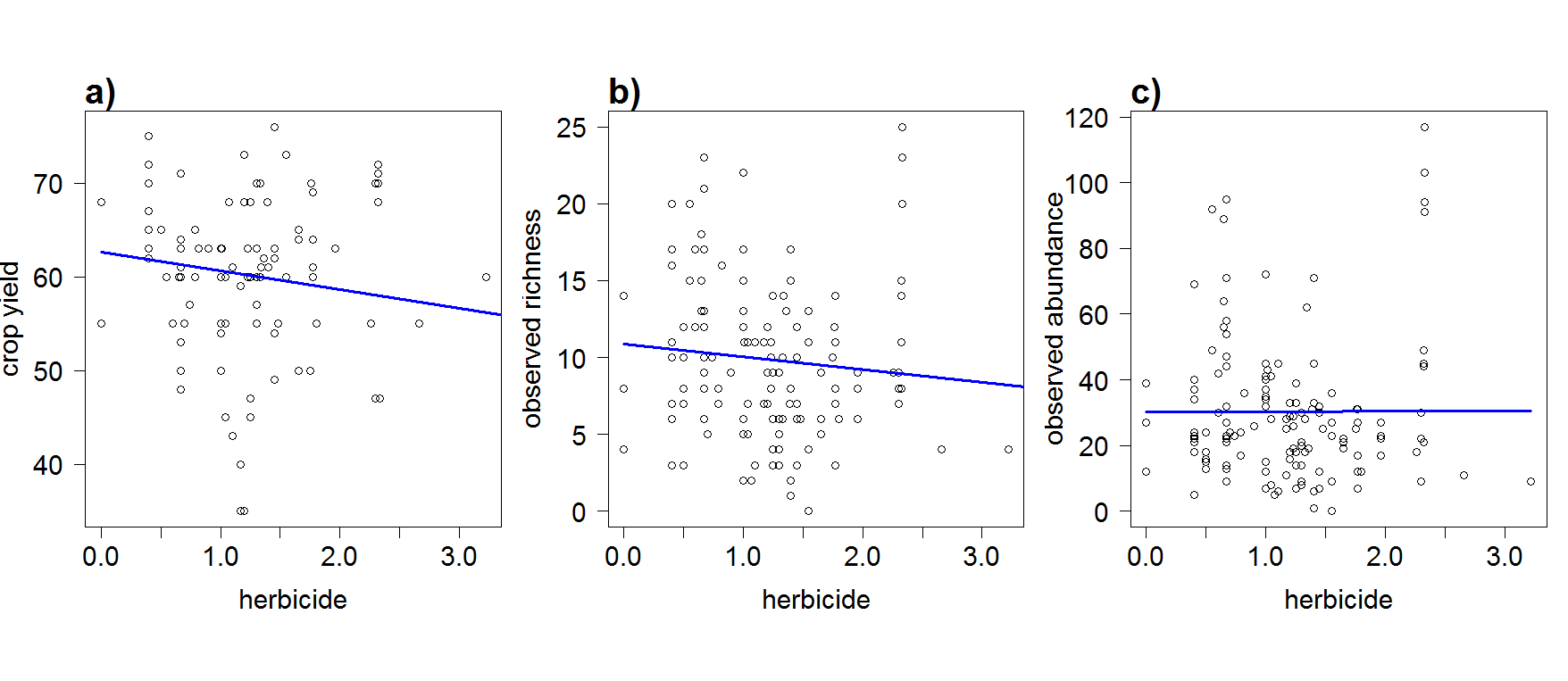


**Figure S3:** **(a) Relationship between observed and predicted weed species richness when herbicide use is represented by TFI.** **The thin and thick lines represent the ‘x-y’ line and the model fit, respectively. (b) Distribution of** η^R^**_Ff_ values estimated when herbicide use is represented by TFI.**

**Figure S4: (a) Expected number of species estimated from simulated data (λ_2_) as a function of the expected number of species estimated from observed data (λ_1_) – R²=0.85, *P*<0.0001. (b) Number of species N_2_ estimated from λ_2_ as a function of the number of species N_1_ estimated from λ_1_ (R²=0.255, *P*<0.0001).**


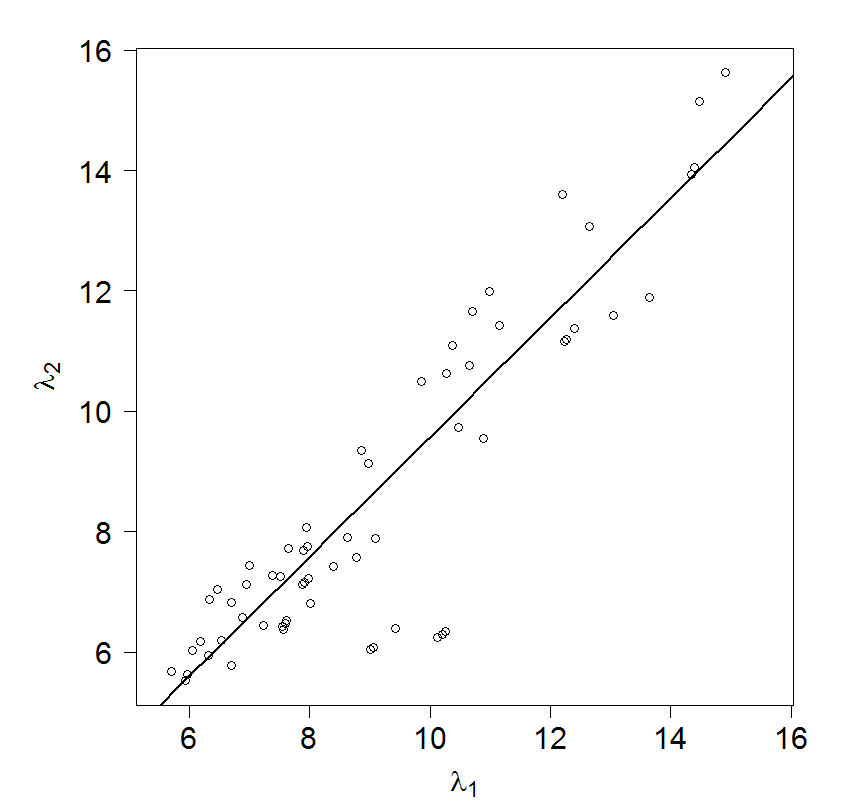

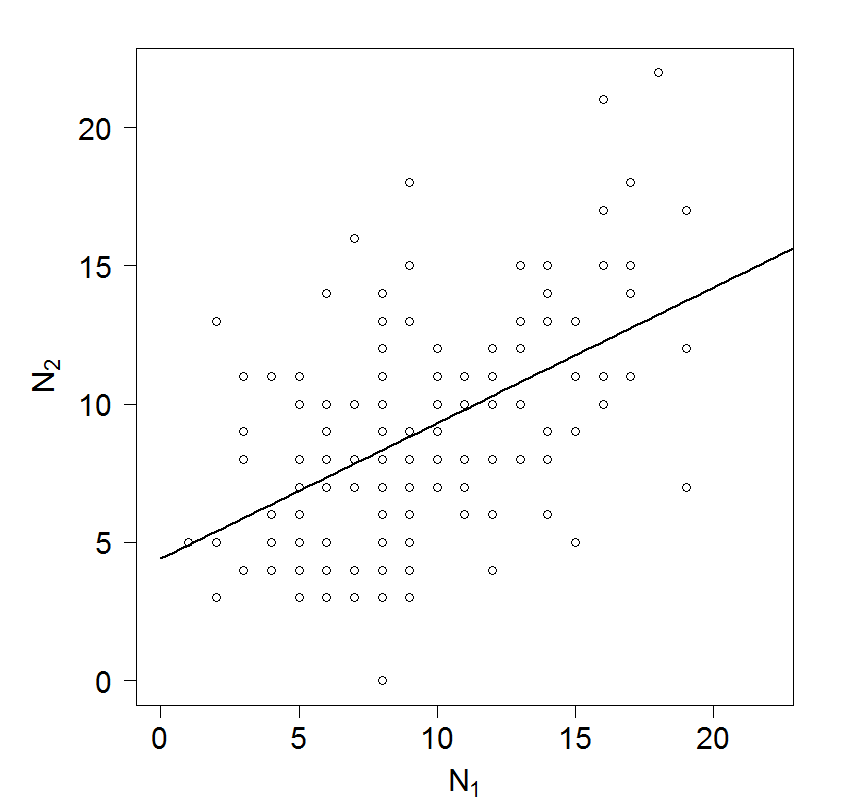


**a** λ_1_

**b** λ_1_

**Figure S5:** η^A^**_Ff_ values estimated when herbicide use is represented by the dose of herbicide as a function of herbicide treatment characteristics: complexity (a), diversity (b) and date of introduction (c). n.s. indicates that the tests (Wilcoxon for a and b, Kruskall-Wallis for c) were not significant.**

**
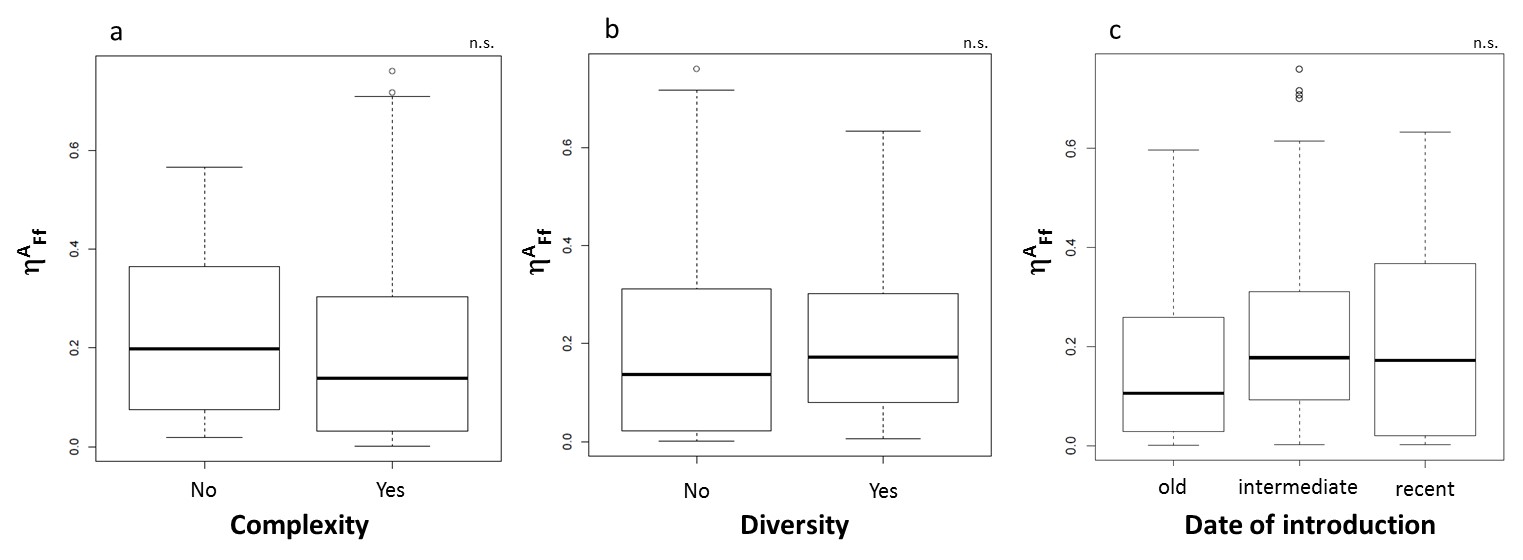
**

**Figure S6: Weed species survival rate depending on the herbicide application rate expressed as the TFI. On the y-axis, weed species are classified from rare to abundant species based on their abundance estimated when no herbicide treatments are applied. Red indicates high mortality rate (survival rate close to 0) and high survival rates are indicated by light yellow or white. The higher mortality rate (red) is associated with the rare species. Four of the most noxious species are: *Veronica persica*, *Galium aparine*, *Alopecurus myosuroides* and *Avena fatua*.**


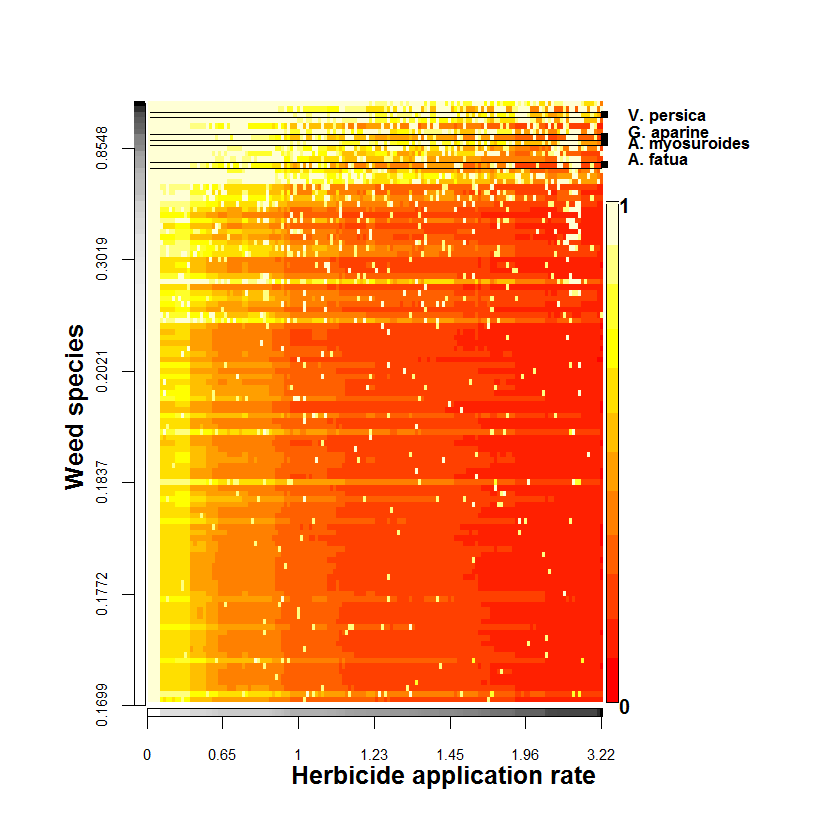


**Table S1:** **General farm characteristics**.

|  | **Field size (ha)** | **Crop yield**  **(q/ha)** | **Herbicide doses (kg/ha)** | **Herbicide TFI** | **Nitrogen Fertilizer (kg/ha)** |
| --- | --- | --- | --- | --- | --- |
| **Average values and ranges** | 4.37  [0.29-23.69] | 60.2  [35 – 76] | 589  [0 - 2252] | 1.262  [0-3.22] | 191.7  [50-385] |

**Table S2: Parameters estimated using observed and simulated data. Parameter µ is the average expected species richness at quadrat level without any treatment. Parameters *a* and *b* describe the effect of the treatment on the weed species richness. 95% confidence intervals are presented between brackets.**

|  | **µ** | **A** | **b** |
| --- | --- | --- | --- |
| **Estimated from observed data** | 14.91  [13.55- 16.42] | 0.78  [0.42-1.34] | 0.14  [0.12-0.18] |
| **Estimated from simulated data** | 14.93  [13.35- 16.76] | 0.73  [0.38-1.26] | 0.15  [0.12-0.18] |

**Table S3: Goodness of fit and parameter estimates for the weed species richness model. DIC values indicate the quality of fit of the model. The smaller the DIC, the better the fit. Parameter µ is the average expected species richness at quadrat level without any treatment. Parameters *a* and *b* describe the effect of the treatment on the weed species richness. 95% confidence intervals are presented between brackets**.

|  | **Without farmer effect** | | **With farmer effect η^R^_F_** | | **With field effect nested in the farmer effect η^R^_Ff_** | |
| --- | --- | --- | --- | --- | --- | --- |
|  | **Dose** | **TFI** | **Dose** | **TFI** | **Dose** | **TFI** |
| **DIC** | 7060 | 7001 | 6121 | 5945 | 5494 | 5525 |
| ***µ*** | 3.48  [3.30-3.70] | 3.52  [3.28-3.79] | 5.92  [5.56-6.30] | 6.08  [5.72-6.49] | 6.55  [5.94-7.04] | 6.30  [5.75-6.79] |
| ***a*** | 0.87  [0.44-1.54] | 0.62  [0.33-1.07] | 3.49  [2.12-5.42] | 3.18  [1.90-4.94] | 1.12  [0.70-1.77] | 2.78  [1.67-4.35] |
| ***b*** | 0.035  [0.027-0.045] | 0.38  [0.25-0.55] | 0.20  [0.18-0.23] | 1.77  [1.17-2.56] | 0.56  [0.45-0.70] | 2.20  [1.54-2.92] |

**Table S4: Goodness of fit and parameter estimates for the weed abundance model. DIC values indicate the quality of fit of the model. The smaller the DIC, the better the fit. Parameter µ is the average expected species abundance at field level without any treatment. Parameters *a* and *b* describe the effect of the treatment on the** weed species abundance. Values between brackets define the 95% confidence interval.

|  | **Without farmer effect** | | **With farmer effect η^A^_F_** | | **With field effect nested in the farmer effect η^A^_Ff_** | |
| --- | --- | --- | --- | --- | --- | --- |
|  | **Dose** | **TFI** | **Dose** | **TFI** | **Dose** | **TFI** |
| **DIC** | 34200 | 33740 | 32590 | 32360 | 31600 | 31630 |
| **µ** | 38.6  [35.2-406] | 41.4  [38.3-43.4] | 41.5  [39.9-43.2] | 42.5  [40.8-44.3] | 43.3  [41.6-45.0] | 43.1  [41.5-44.8] |
| ***a*** | 397.9  [21.1-611.4] | 111.4  [46.5-157.3] | 73.1  [49.8-105.1] | 39.1  [27.0-55.8] | 5.05  [3.08-7.94] | 12.44  [7.46-19.71] |
| ***b*** | 0.222  [0.21-0.30] | 0.556  [0.50-0.65] | 0.372  [0.35-0.39] | 0.985  [0.88-1.11] | 0.81  [0.70-0.94] | 1.90  [1.52-2.39] |

**Table S5: List of the active ingredients used by the 30 farmers in the study area in 2011.**

| **Active ingredients** |
| --- |
| 2,4-MCPA |
| Amidosulfuron |
| Bromoxynil phénol |
| Carfentrazone-Ethyle |
| Clodinafop-propargyl |
| Clopyralid |
| Cloquintocet-mexyl |
| Diclofop-méthyl |
| Diflufénicanil |
| Florasulam |
| Fluroxypyr |
| Glyphosate |
| Iodosulfuron-méthyl-sodium |
| Isoproturon |
| Mécoprop(-P) |
| Mésosulfuron-méthyl |
